# Elastographic Evaluation of the Adrenal Glands of Dogs With Hypercortisolism

**DOI:** 10.1111/vru.70156

**Published:** 2026-03-19

**Authors:** Fernanda de Paula Sesti, Gabriel Marchiori Gonzaga, Bruno Alberigi

**Affiliations:** ^1^ Programa de Pós Graduação em Medicina Veterinária (PPGMV), Patologia e Ciências Clínicas Universidade Federal Rural do Rio de Janeiro Seropédica Rio de Janeiro Brazil; ^2^ Departamento de Medicina e Cirurgia Veterinária Instituto de Veterinária Universidade Federal Rural do Rio de Janeiro Seropédica Rio de Janeiro Brazil

**Keywords:** adrenal hyperplasia, adrenal neoplasms, veterinary ultrasonography

## Abstract

Elastography is a promising technique for assessing tissue stiffness in the adrenal glands of dogs with hypercortisolism (HC). This study compared 30 dogs, 15 of which were healthy (control group) and 15 diagnosed with HC, confirmed by low‐dose dexamethasone suppression test (*n* = 11) or ACTH stimulation test (*n* = 4) without prior treatment. Ultrasound measurements revealed a significant increase in the dimensions of the adrenal glands, especially in the left adrenal gland, with more frequent changes in the cranial pole (86.7%, *p* = 0.00003) and caudal pole (80%, *p* = 0.00005). Qualitative elastography indicated varied tissue stiffness patterns in sick dogs, with a predominance of mixed patterns (46.7%), whereas dogs in the control group showed uniform moderate stiffness. Semiquantitative analysis showed that the adrenal glands of sick dogs were significantly stiffer compared to the adjacent mesentery, with variations ranging from 33% to 80% stiffer. The Mann–Whitney test revealed statistically significant differences in adrenal stiffness between the groups (*U* = 4.500; *Z* = −4.621; *p* < 0.001). These findings suggest that elastography, combined with conventional ultrasonography, may be an effective complementary diagnostic tool in detecting adrenal changes in dogs with HC.

**Abbreviations**
BCS: body condition score
CEUA/IV/UFRRJ: Ethics Committee on the Use of Animals of the Veterinary Institute of the Federal Rural University of Rio de Janeiro
CI: confidence intervals
HC: hypercortisolism
ICF: informed consent form
LAG: left adrenal gland
L‐Ref Ratio: liver to reference ratio
PD: polydipsia
PF: polyphagia
PU: polyuria
RAG: right adrenal gland
USG: abdominal ultrasound

## 1 Introduction

Hypercortisolism (HC) is characterized by chronic elevation of serum cortisol levels [1, 2]. It can be classified as ACTH‐dependent when there is an elevation in corticotropin levels related to pituitary neoplasms and ACTH‐independent in patients with adrenal neoplasms [3, 4, 5].

About 85% of HC cases are ACTH‐dependent and occur due to the presence of small adenomas in the pituitary gland measuring about 3 mm in diameter or macroadenomas that can measure 3 to 10 mm in diameter [6, 7].

Abdominal ultrasound (USG) helps in the evaluation of the adrenal glands, as it can estimate their dimensions and observe changes in the shape, echotexture, and echogenicity of the parenchyma [8, 9]. Recent studies, such as that by Melián et al. [10], have proposed new upper limits for the height of the cranial poles and tails of the adrenal glands in clinically healthy dogs, highlighting the importance of considering ideal body weight when interpreting ultrasound measurements.

In association with ultrasonography, the elastography technique can be used to analyze tissue stiffness through mechanical excitation in a given tissue [11]. Tissue elasticity is assessed by the force of the pressure applied and the degree of deformation presented [12].

The colors displayed determine the degree of stiffness, with dark areas observed in stiff tissues and light areas in softer tissues [13]. Each device has a color scale [14].

In humans, elastographic evaluation of the adrenal glands is used and is viable for the identification of adrenal neoplasms and is effective in characterizing malignant and benign lesions [15]. A study by Fernandez et al. [16] presented promising results for evaluation using elastography in healthy dogs. The evaluation was performed without difficulty, and qualitative analysis showed that the adrenal parenchyma is not deformable and has a homogeneous appearance.

Sesti et al. [17] evaluated the tissue stiffness of the adrenal glands in healthy dogs and compared it with that of the adjacent mesentery. Qualitative and semiquantitative analysis revealed similar stiffness between the structures, indicating that elastography may be a useful tool for assessing the integrity of the adrenal glands, using the mesentery as a reference.

Elastographic analysis is performed safely and noninvasively and provides information on the tissue characteristics of the evaluated structure [18, 19]. The results obtained aid in the detection of fibrotic processes or possible neoplastic infiltrations present in the examined organ, providing important information for patient follow‐up and prognosis [20].

Sesti et al. [21] reported a clinical case of a West Highland White Terrier dog that presented adrenal neoformation during USG characterized by parenchymal heterogeneity. Elastography complemented the evaluation by indicating high tissue stiffness in the lesion, suggesting possible malignancy. The diagnosis of adrenocortical carcinoma was confirmed after histopathology.

The objective of this study was to investigate the tissue stiffness of the adrenal glands of dogs newly diagnosed with untreated HC, whether ACTH‐dependent or ACTH‐independent, using qualitative and semiquantitative elastography.

## 2 Materials and Methods

This study was to the Animal Use of the Veterinary Institute of the Federal Rural University of Rio de Janeiro (CEUA/IV/UFRRJ), under protocol number 7099080423, and certified according to the criteria for the use of animal experimentation. Only dogs whose owners agreed to the proposed procedures and signed the Free and informed consent form (TCLE) were included.

The study was conducted at the Small Animal Veterinary Hospital of the Federal Rural University of Rio de Janeiro. Fifteen dogs recently diagnosed with ACTH‐dependent or ACTH‐independent HC, without prior treatment with trilostane or mitotane, weighing between 2.5 and 35.0 kg, were included. Fifteen other healthy dogs formed the control group. In total, 60 adrenal glands were evaluated, 30 from healthy dogs and 30 from sick dogs treated and referred from the small animal medical clinic sector of the HVPA‐UFRRJ.

To eliminate the influence of the wide variation in body weight observed among the dogs included and to standardize the interpretation of ultrasound measurements, the animals were classified into three categories of body size, as described by Melián et al. [10] and Soulsby et al. [22]: small (<10 kg), medium (10–25 kg), and large (>25 kg). All adrenal measurements were analyzed according to the specific normal ranges for each weight group to avoid direct comparisons between dogs of different sizes. Thus, the evaluation of adrenal gland dimensions was performed within the category corresponding to each dog, ensuring that the interpretation was proportional to its body size. In addition, statistical analyses were conducted with adjustment for body weight, reducing the impact of this variable on the results and ensuring that the differences identified between the groups were not solely due to size variation. Thus, the influence of body weight was controlled both methodologically and statistically, allowing for an adequate comparison between the groups studied.

Age was not used as a matching criterion between groups, as this was a consecutive clinical sample. Age was recorded for all dogs and compared between groups in the statistical analysis, being considered a potential confounding variable. To ensure adequate characterization of the control group, only dogs considered clinically healthy based on multiple complementary criteria were included. All animals underwent a complete physical examination, detailed medical history, and comprehensive laboratory evaluation, including complete blood count and serum biochemistry, namely, aspartate aminotransferase, alanine aminotransferase, alkaline phosphatase, cholesterol, triglycerides, and serum glucose.

Only dogs without clinical or laboratory abnormalities were eligible for the control group. Additionally, animals with a history of glucocorticoid use were excluded, as well as those with clinical signs compatible with endocrinopathies, such as polyuria, polydipsia, polyphagia, symmetrical bilateral alopecia, abdominal distension, or exercise intolerance. Abdominal ultrasonography was also used as a mandatory criterion for confirming adrenal normality, and only dogs without structural changes in the glands were included.

In cases where abdominal masses were identified, their adrenal origin was confirmed by specific ultrasonographic criteria, including anatomical contiguity between the lesion and the gland, typical topographic location, cranial to the right kidney or medial to the left kidney, and partial preservation of the adjacent cortex or medulla. Only masses that met all these criteria were classified as adrenal and included in the analysis. Dogs with HC were diagnosed by means of low‐dose dexamethasone suppression or ACTH stimulation tests, with measurements of basal cortisol and after 4 and 8 h.

The dogs were fasted for 8 h prior to evaluation and then assessed by USG using a Vinno V5 Vet device equipped with linear (6–18 MHz) and microconvex (4–12 MHz) multifrequency transducers. The choice of transducer followed a standardized protocol based on the size of the animal: Small dogs were preferably evaluated with the linear transducer, due to its higher spatial resolution, whereas medium and large dogs were examined with the microconvex transducer, which offers better penetration without significant loss of image quality. This standardization aimed to reduce differences related to depth and level of detail visualized between animals of different sizes.

All examinations were performed by a single veterinarian specializing in diagnostic imaging, with 5 years of experience, ensuring technical uniformity and minimizing interobserver variations. To control interobserver variability, each measurement was repeated five times per adrenal gland, considering only sequences with a stable compression cycle and the absence of artifacts.

The ultrasound evaluation followed a previously defined protocol, including longitudinal and transverse scanning of the adrenal glands, with analysis of shape, contours, echogenicity, echotexture, cortical and medullary thickness, relationship between layers, and identification of nodules or neoformations, in addition to evaluation of the adjacent mesentery, as described by Soulsby et al. [22]. Measurements of the height of the cranial and caudal poles and the length of the adrenal glands were performed strictly following the criteria of Melián et al. [10], using specific normal ranges by body size for the detection of adrenomegaly.

No lower and upper limits were established for the measurements of the cranial and caudal poles of the adrenal glands according to body weight. The measurements were analyzed descriptively, with the aim of presenting the individual values of adrenal dimensions in clinically healthy dogs and sick dogs. Reference ranges were not defined due to the limited sample size and heterogeneity of body weights, which was not the objective of the present study.

After the B‐mode examination, qualitative compressive elastography of the adrenal glands was performed using the same equipment and transducers. The method is based on a color scale ranging from red (softer tissues) to blue (stiffer tissues). Semiquantitative analysis was performed by comparing the stiffness of the adrenal gland with the adjacent mesentery or healthy adrenal tissue in cases of neoformations. Each gland was evaluated under slight mechanical pressure from the transducer, and five measurements were taken per adrenal gland to assess whether the compression cycles and data acquisition ruler were performed successfully, according to Feliciano et al. [20].

In all dogs, the adjacent mesentery was used as reference tissue for elastographic analyses. Comparisons with apparently preserved adrenal parenchyma were not used as a reference, as subclinical histopathological changes cannot be ruled out in glands affected by HC.

Qualitative results were classified on a scale of 1 to 4, based on the stiffness observed 1—Adrenal gland with preserved stiffness (normal), 2—mixed stiffness pattern, 3—moderate stiffness (gland 65% stiffer), and 4—high stiffness (gland 95% stiffer).

Semiquantitative analysis was performed using the equipment software, which presents stiffness as a percentage, comparing it with the adjacent mesentery as reference tissue. A value of 100% (liver to reference ratio [L‐Ref Ratio] 1.00—ratio between the stiffness of the altered tissue and healthy tissue) indicates stiffness identical to the adjacent mesentery as reference tissue, whereas 0% (L‐Ref Ratio 0.00) reflects stiffness 100% higher than that of the adjacent mesentery as reference tissue.

All examinations were performed by a veterinarian specializing in diagnostic imaging. The professional received prior training to ensure standardization of elastographic analysis criteria and techniques. All decisions related to data recording and analysis were based on criteria defined by Fernandez et al. [16]. The technical parameters used in the examinations included adrenal gland size, ultrasound morphology and architecture, and qualitative and semiquantitative elastographic assessment.

The determination of the adjusted sample size was used only as a methodological reference and is not a central part of the statistical analysis presented. For comparisons between groups, the Proportions, Chi‐Square, or Fisher's Exact tests were applied to categorical variables, whereas the Student *t*‐test and Mann–Whitney test were used for parametric and nonparametric variables, respectively. The correlation between nonparametric variables was assessed using Spearman's test. The results were expressed as mean ± standard deviation and 95% confidence intervals (95% CI). The level of significance adopted was *p* ≤ 0.05. All analyses were performed using SPSS software version 29.0.1.

## 3 Results

The study analyzed a total of 30 dogs, divided equally into two groups: 15 healthy dogs, which comprised the control group, and 15 dogs diagnosed with HC, classified as the sick group. The overall mean age was 8.80 ± 3.56 years, ranging from 2 to 15 years, with dogs in the diseased group being significantly older than those in the control group (9.47 ± 2.41 years vs. 7.73 ± 3.97 years, *p* = 0.0080).

Of the 30 dogs evaluated in the study, the majority were females, totaling 21/30 animals (70%), whereas males represented 9/30 individuals (30%). In the group of dogs diagnosed with the disease, 10/15 animals (66.7%) were females, and 5/15 (33.3%) were males. In the control group, the predominance of females was also evident, with 11/15 dogs (73.3%) belonging to this sex, whereas males corresponded to 4/15 individuals (26.7%).

Of all the dogs evaluated, the breed distribution was diverse. Mixed‐breed dogs represented the largest portion, with 10/30 animals (33.3%), followed by Shih Tzu, with 6/30 individuals (20%), and Poodle, with 4/30 (13.3%). Yorkshire Terriers accounted for 3/30 dogs (10%), whereas Pinschers, Lhasa Apsos, Border Collies, Dachshunds, Giant Poodles, and Tenerife dogs had 1/30 representative each (3.3%).

In the group of sick dogs, consisting of 15 individuals, mixed breeds and Poodles were equally prevalent, with 3/15 dogs each (20%). Shih Tzus contributed 2/15 dogs (13.3%), as did Yorkshire Terriers (13.3%). In addition, the Lhasa Apso, Border Collie, Dachshund, Giant Poodle, and Tenerife breeds had 1/15 sick representatives each (6.7%).

Among healthy dogs, which also totaled 15 animals, mixed breeds predominated widely, with 7/15 representatives (46.7%), followed by Shih Tzu, with 4/15 (26.7%). Poodles, Yorkshire Terriers, Pinschers, and Lhasa Apsos contributed 1/15 healthy animals each (6.7%).

The average weight of the dogs was 11.15 ± 7.45 kg, ranging from 2.70 to 33.90 kg. Although the difference in weight between the groups was not statistically significant (*p* = 0.1704), the sick group had a higher concentration of animals weighing between 8 and 12 kg, whereas the control group showed greater variability.

Hormonal evaluation confirmed adrenal dysfunction in sick dogs. Low‐dose dexamethasone suppression was performed in 11/15 dogs and ACTH stimulation in 4/15 dogs. In basal cortisol, 46.7% had altered values, whereas in post‐dexamethasone, 73.3% showed inadequate suppression. Post‐ACTH cortisol also revealed alterations in 73.3% of cases, with values ranging from 18.5 to 26.75 µg/dL. These findings indicate exacerbated adrenal activity in sick dogs.

The dimensions of the adrenal glands, assessed by ultrasonography, also showed significant differences between the groups. In the control group (Table 1), adrenal measurements were within normal limits, whereas in the sick group (Table 2), increases were observed, especially in the left adrenal gland (LAG).

**TABLE 1:** Body weight and height of the cranial and caudal poles of the left (LAG) and right (RAG) adrenal glands in healthy dogs.

Weight (kg)	Height of the caudal pole LAG (cm)	Cranial pole height LAG (cm)	RAG caudal pole height (cm)	Height of RAG cranial pole (cm)
3.0	0.44	0.36	0.43	0.47
4.00	0.42	0.37	0.45	0.44
5.40	0.35	0.40	0.47	0.45
6.00	0.51	0.43	0.41	0.50
6.00	0.49	0.47	0.46	0.53
6.00	0.57	0.51	0.54	0.57
6.70	0.46	0.38	0.35	0.41
7.00	0.50	0.46	0.58	0.52
7.00	0.50	0.46	0.39	0.50
7.70	0.53	0.49	0.69	0.53
11.00	0.58	0.49	0.56	0.52
13.30	0.57	0.52	0.55	0.53
15.00	0.66	0.64	0.75	0.70
20.00	0.68	0.60	0.65	0.66
22.00	0.79	0.64	0.75	0.70

Abbreviations: LAG, left adrenal gland, RAG right adrenal gland.

**TABLE 2:** Body weight and height of the cranial and caudal poles of the left (LAG) and right (RAG) adrenal glands in healthy dogs.

Weight (kg)	Height of the caudal pole LAG (cm)	Height of the cranial pole LAG (cm)	Height of caudal pole RAG (cm)	Height of RAG cranial pole (cm)
2.70	0.70	0.81	1.16	1.18
5.80	1.35	0.77	0.95	0.85
6.00	0.91	0.51	0.97	0.56
6.80	0.64	0.93	0.49	0.66
8.00	2.66	1.69	3.94	1.83
8.35	0.85	0.79	0.62	0.77
10.00	0.72	0.97	0.49	0.64
10.00	0.86	2.70	0.61	1.22
10.00	0.58	0.45	0.53	0.70
12.00	0.83	0.75	0.64	0.85
12.40	0.62	0.75	0.76	0.60
21.90	0.89	0.98	1.17	1.26
22.60	0.92	0.70	1.02	0.82
24.00	0.83	0.98	0.62	0.99
33.90	2.83	3.40	2.29	2.41

At the cranial pole of the LAG (86.7%), changes were more frequent (*p* = 0.00003), followed by the caudal pole of the same gland (80%) (*p* = 0.00005). In the right adrenal gland (RAG), the changes were less pronounced but still statistically significant, with *p* = 0.0004 for the caudal pole (53.3% of dogs) and *p* = 0.002 for the cranial pole (60% of dogs).

Elastography revealed significant qualitative differences in adrenal stiffness between the groups evaluated. The Mann–Whitney test revealed statistically significant differences between the groups analyzed. The stiffness of the RAG was *p* < 0.001, and for RAG stiffness, *p* < 0.001, indicating significant variations in the distributions of both variables between groups.

**FIGURE 1:**
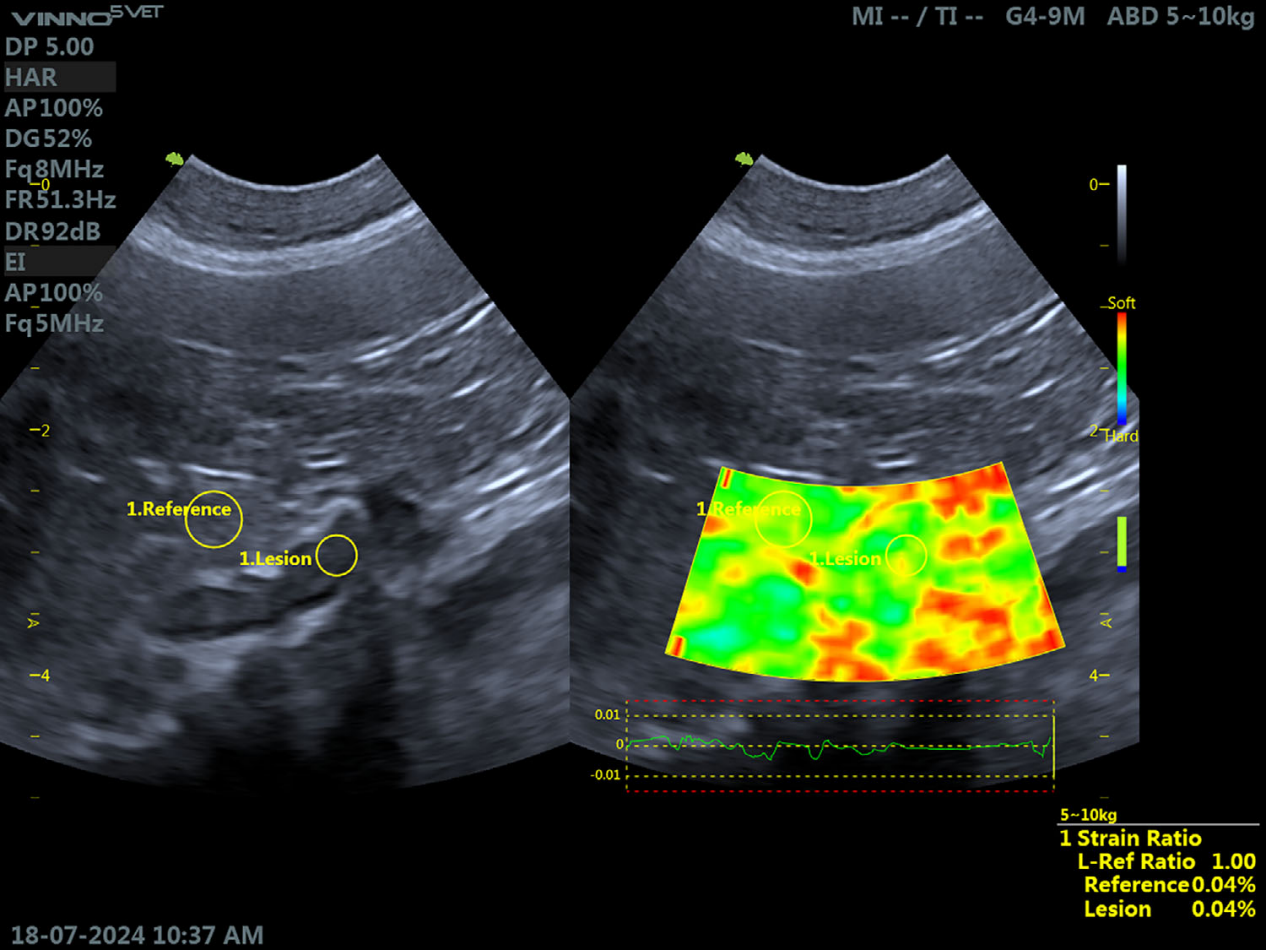
Elastographic evaluation of the left adrenal gland (LAG), in longitudinal section, and the adjacent mesentery in a clinically healthy dog. The examination was performed with a microconvex transducer, frequency of 8 MHz, gain of 52%, and depth of 5 cm. Mixed‐breed dog, female, 5 years old, and 7 kg body weight. On the left, a B‐mode ultrasound image shows the delimitation of the adrenal gland and the region of interest in the adjacent mesentery, used as reference tissue. On the right, a qualitative elastographic image showing moderate tissue stiffness, with a similar color pattern between the adrenal gland and the reference tissue. Semiquantitative analysis by strain ratio, using the adjacent mesentery as reference tissue, showed equivalent values (reference/lesion ratio = 1.00). *Source*: Personal archive.

In the control group, all adrenal glands (100%) showed predominantly green staining, indicating moderate stiffness and characteristics similar to the adjacent mesentery (Figure 1).

In contrast, in the group of sick dogs, a higher prevalence of mixed staining patterns was observed (46.7%). The categories of high rigidity and preserved rigidity had similar frequencies, with 20% of cases each, whereas moderate rigidity was the least frequent, corresponding to only 13.33% of cases. These results indicate a statistically significant predominance of the mixed pattern in the diseased group, with a relatively balanced distribution between normal and high rigidity, and a lower occurrence of moderate rigidity.

In the semiquantitative assessment of adrenal gland stiffness in sick dogs compared to the adjacent mesentery, a heterogeneous distribution of results was observed, as shown in Table 3.

**TABLE 3:** Distribution of adrenal gland stiffness in sick dogs in relation to the adjacent mesentery, assessed by elastography.

Stiffness in relation to the adjacent mesentery	Proportion (%)	Absolute number (*n*)
Equal to mesentery (100%)	20.0	6
Stiffer than the mesentery (67%)	20.00	6
Stiffer than the mesentery (33%)	16.67	5
Stiffer than the mesentery (75%)	13.3	4
Stiffer than the mesentery (25%)	10.00	3
Stiffer than the mesentery (50%)	6.67	2
Stiffer than the mesentery (60%)	6.67	2
Stiffer than the mesentery (80%)	3.33	1
Stiffer than the mesentery (40%)	3.33	1

During the evaluation of dogs diagnosed with HC, adrenal neoplasms were observed in different locations, as described in Table 4.

**TABLE 4:** Distribution of adrenal neoplasms in dogs diagnosed with hypercortisolism. LAG ‐ left adrenal gland; RAG ‐ right adrenal gland.

Characteristic	Proportion	Absolute number (*n*)
Neoplasm only in LAG	13.33	2
Neoformation only in RAG	13.33	2
Bilateral adrenal neoplasm	26.67	4
Total	53.3	8

**FIGURE 2:**
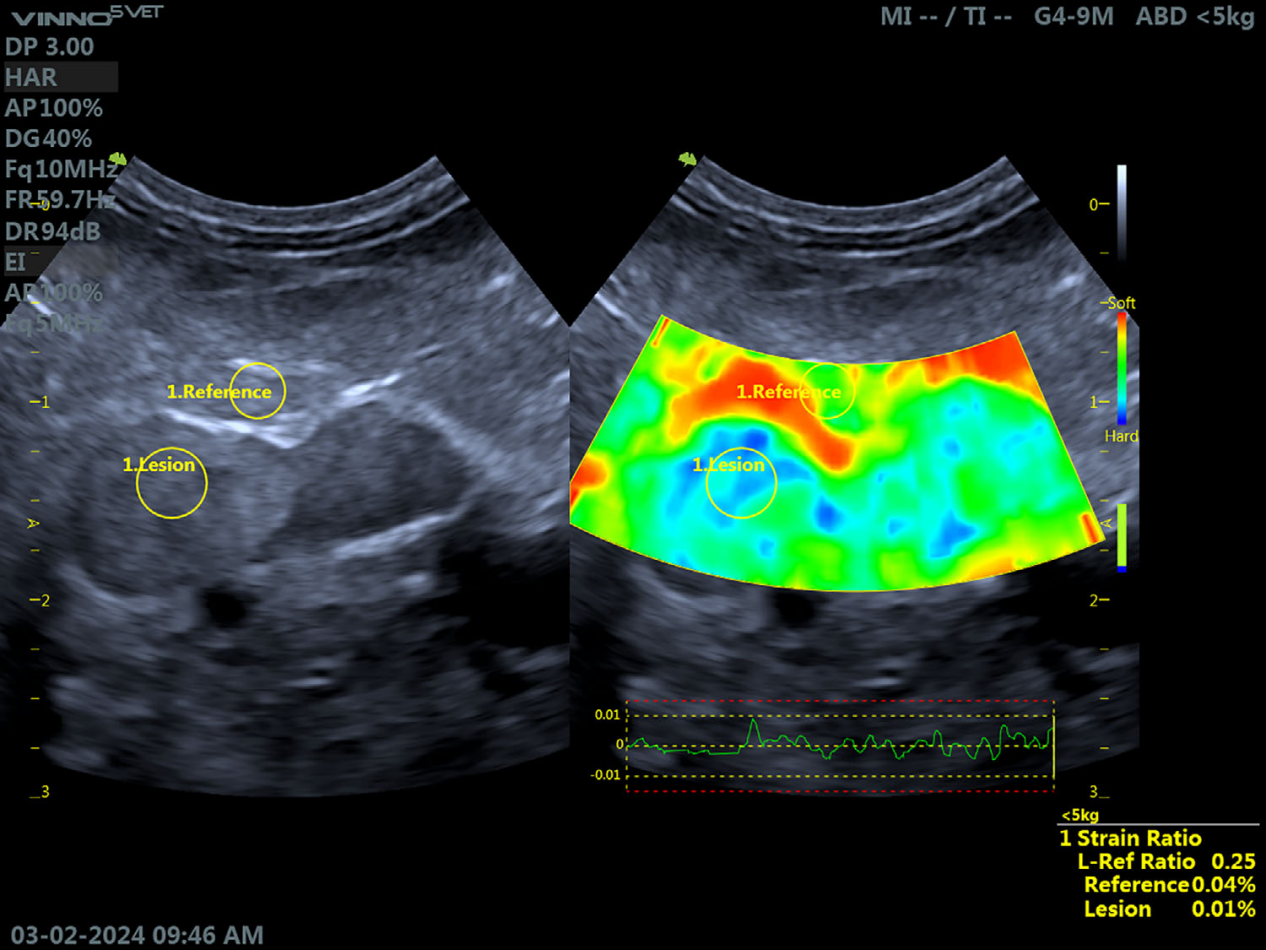
Elastographic evaluation of the left adrenal gland (LAG), in longitudinal section, and the adjacent mesentery in a dog diagnosed with hypercortisolism (HC). A nodular lesion is observed located at the cranial pole of the adrenal gland. The examination was performed with a microconvex transducer, frequency of 10 MHz, gain of 40%, and depth of 3 cm. Female Poodle, 12 years old, weighing 8 kg. On the left, a B‐mode ultrasound image shows the delimitation of the left adrenal gland, the nodular lesion in the cranial pole, and the region of interest in the adjacent mesentery, used as reference tissue. On the right, a qualitative elastographic image showing increased tissue stiffness of the adrenal cranial pole in relation to the adjacent mesentery, used as reference tissue, with a predominance of blue areas in the adrenal gland when compared to the reference tissue. Semiquantitative analysis by strain ratio, using the adjacent mesentery as reference tissue, showed greater stiffness of the adrenal cranial pole (reference/lesion ratio = 0.25). *Source*: Personal archive.

Of these, 1/8 (12.5%) presented 75% greater stiffness of the LAG neoformation compared to the adjacent mesentery (Figure 2). In addition, 1/8 (12.5%) presented 67% greater stiffness of the LAG neoformation compared to healthy parenchyma and the adjacent mesentery.

Among dogs with identified neoformation in the RAG, two distinct patterns of tissue stiffness were observed. Of these, 1/8 (12.5%) had RAG neoplasm stiffness 33% higher than the adjacent mesentery. In addition, 1/8 (12.5%) had ADE neoplasm stiffness 67% higher than the adjacent mesentery.

Only 1/8 (12.5%) of dogs had bilateral adrenal neoplasms with healthy tissue for comparison. The neoplasm in the LAG had 50% greater stiffness than the mesentery. In the neoplasm present in the RAG, 75% greater stiffness than the adjacent mesentery was identified.

Among dogs with total bilateral adrenal neoplasia, three dogs (37.5%) were found to have a complete absence of healthy adrenal tissue in the LAG and RAG. Thus, tissue stiffness was compared exclusively with the adjacent mesentery, as described in Table 5.

**TABLE 5:** Tissue stiffness patterns in dogs with total bilateral adrenal neoformation. LAG ‐ left adrenal gland; RAG ‐ right adrenal gland

Characteristic	Proportion (%)	Absolute number (*n*)
LAG 80% stiffer and RAG 75% stiffer than the mesentery	33.3	1
LAG and RAG 67% stiffer than the mesentery	33.3	1
LAG 50% stiffer and RAG 75% stiffer in relation to the mesentery	33.3	1

Analysis of tissue stiffness patterns in dogs revealed a higher prevalence of patterns equal to the mesentery and 67% stiffer, which together accounted for 42.86% of observations. Intermediate values, such as 33% and 75% stiffer, corresponded to 28.58%, whereas less frequent patterns, such as 80% and 40% stiffer, accounted for only 3.57% each. In cases of bilateral neoformations, the patterns showed uniform frequencies of 33.3% per category. The cumulative analysis highlighted the predominance of more frequent patterns in general cases and a balanced distribution in bilateral conditions, reflecting the diversity and complexity of the tissue changes observed.

## 4 Discussion

The older age of hypercortisolemic animals is within expectations, as previous studies associate the prevalence of HC with middle‐aged to older animals [7, 23]. The predominance of mixed‐breed dogs is consistent with the profile of the population served by the veterinary hospital, located in a low‐income region. Although mixed‐breed dogs represented the majority of the studied population, the highest frequency of HC cases was observed in Shih Tzu and Poodle breeds, which may be related to a breed predisposition previously described in the literature [24].

Hormonal evaluation confirmed adrenal dysfunction in the dogs in this study, with inadequate suppression after dexamethasone in 73.3% of cases and changes in post‐ACTH cortisol in the same proportion. These findings corroborate the presence of exacerbated adrenal activity, characteristic of HC, and highlight the high sensitivity of low‐dose dexamethasone suppression for detecting the disease, as previously described [2,^25^,10]. These results reinforce the importance of hormonal evaluation as a diagnostic tool in dogs with clinical signs suggestive of HC.

Unlike what was described by Soulsby et al. [22], who identified the caudal pole of the left adrenal gland as the main site of dimensional variation in healthy dogs, the results of the present study demonstrated a predominance of changes in the cranial pole of the left adrenal gland in dogs with HC. This finding suggests that, under pathological conditions, adrenal enlargement does not follow the physiological growth pattern observed in healthy animals and may reflect the presence of hyperplastic and/or degenerative processes with asymmetric distribution throughout the gland. Thus, ultrasonographic evaluation of both adrenal poles is essential for the adequate detection of changes associated with the disease.

Unlike the findings of Soulsby et al. [22], who identified the caudal pole of the LAG as the main affected area in healthy dogs, our findings show a predominance of changes in the cranial pole of the LAG in dogs with HC. This suggests that, in the disease, hyperplastic and degenerative processes affect the gland in a way that is distinct from physiologic growth, highlighting the importance of evaluating both poles on ultrasound.

Although the amount of surrounding fat could theoretically influence elastographic measurements, the animals in this study were not grouped according to body condition score, which is a methodological limitation. Nevertheless, elastography consistently demonstrated differences in adrenal gland tissue stiffness between the groups evaluated. The glands of healthy dogs showed moderate stiffness, similar to that of the adjacent mesentery, whereas the glands of dogs with HC predominantly exhibited mixed patterns, followed by high stiffness. Considering that the elastographic evaluation was performed comparatively with the adjacent reference tissue of the animal itself, it is unlikely that individual variation in periadrenal fat alone explains these findings. Thus, the results reflect structural changes associated with adrenal hyperplasia and the presence of neoplasms, as previously described [8, 16, 21], reinforcing the potential of elastography as a complementary diagnostic tool in veterinary medicine.

In cases of adrenal neoplasms, tissue stiffness was significantly higher compared to normal glands, which was expected, as these patterns reinforce the usefulness of elastography in characterizing adrenal tissues and differentiating between hyperplasia and neoplasms described in studies that evaluated different tissues [17, 19]. Recent studies also associate high stiffness patterns with neoplastic infiltrations [21].

Elastographic characterization in healthy dogs revealed homogeneous patterns and moderate stiffness, as described in previous studies [16, 18]. Deviations from these patterns were consistently associated with pathological changes, highlighting the diagnostic value of elastography in adrenal glands with suspected HC. These results reinforce that elastography has great potential to complement the information obtained by conventional ultrasound, providing additional information on tissue elasticity that may aid in the clinical management of patients with HC.

Despite the promising findings, some limitations of the present study should be considered. Histopathological validation of the adrenal glands evaluated was not performed, as none of the dogs included in the study underwent adrenalectomy due to restrictions imposed by their owners. Consequently, it was not possible to histologically confirm the nature of the structural changes observed, making it impossible to definitively differentiate between hyperplastic and neoplastic processes, although histopathology is considered the gold standard for this distinction. Thus, interpretations were based on clinical, laboratory, and imaging findings. In addition, the relatively small sample size may limit the generalization of the results. Future studies involving larger samples and histopathological validation will be essential to confirm the findings and refine the applicability of adrenal elastography in clinical practice.

On the basis of the results obtained, this study demonstrates that qualitative and semiquantitative elastography is a promising tool for assessing structural changes in the adrenal glands of dogs with HC. Significant differences in tissue stiffness between healthy and diseased groups suggest that the technique may be useful in characterizing hyperplasia and neoplasms. However, future studies, including histopathological validation, are essential to consolidate the clinical application of elastography in veterinary practice.

## 5 Conclusion

The present study demonstrated that elastography, both qualitative and semiquantitative, is a useful method for evaluating the adrenal glands in dogs with HC. Consistent differences in tissue stiffness were identified between the glands of healthy dogs and dogs with HC, reflecting structural changes associated with the disease. These results indicate that elastography can act as a complementary tool to conventional ultrasonography in adrenal evaluation in dogs.

## Author Contributions

Data collection: Fernanda de Paula Sesti and Gabriel Marchiori Gonzaga. Data analysis and interpretation: Fernanda de Paula Sesti and Bruno Alberigi. Article prepared by: Fernanda de Paula Sesti, Gabriel Marchiori Gonzaga, and Bruno Alberigi. Review of the article: Fernanda de Paula Sesti and Bruno Alberigi. Final approval: Fernanda de Paula Sesti, Gabriel Marchiori Gonzaga, and Bruno Alberigi.

## Disclosure

Preliminary results from this study were presented at the CIABEV 2024 as an abstract, but the full dataset, analysis, and discussion have not been published or submitted elsewhere. F. P. Sesti, G. M. Gonzaga, M. Damatis, and B. Alberigi., “Elastographic Evaluation of the Adrenal Glands of Healthy Dogs,” Acta Scientiae Veterinariae 2, no.51 (2024): 10 (a). F. P. Sesti, G. M. Gonzaga, F. M. T. M. Zimmer, T. V. D. S. Cruz, and B. Alberigi, “Adrenocortical Carcinoma in a West White Terrier: Clinical and Diagnostic Approach,” Brazilian Journal of Veterinary Medicine 46 (2024): e005424. 10.29374/2527‐2179.bjvm005424 (b).

## Conflicts of Interest

The authors declare no conflicts of interest.

## Dissemination of Previous Presentations or Publications

F. P. Sesti, G. M. Gonzaga, M. Damatis, and B. Alberigi, “Elastographic Evaluation of the Adrenal Glands of Healthy Dogs”. *Acta Scientiae Veterinariae* 2, no. 51 (2024): 10 (a). F. P. Sesti G. M. Gonzaga, F. M. T. M. Zimmer, T. V. D. S. Cruz, and B. Alberig, “Adrenocortical Carcinoma in a West White Terrier: Clinical and Diagnostic Approach,” *Brazilian Journal of Veterinary Medicine* 46 (2024): e005424. 10.29374/2527‐2179.bjvm005424 (b).

## Data Availability

The data that support the findings of this study are available from the corresponding author upon reasonable request.
